# Supplementary material for: DNA damage induced by CDK4 and CDK6 blockade triggers anti-tumor immune responses through cGAS-STING pathway
Source: Commun Biol. 2023 Oct 13;6:1041. doi: 10.1038/s42003-023-05412-x (PMC10575937; doi:10.1038/s42003-023-05412-x)
Supplement: Supplementary file 2 — Supplementary Information [file 42003_2023_5412_MOESM2_ESM.pdf]

Supplementary Figure 1

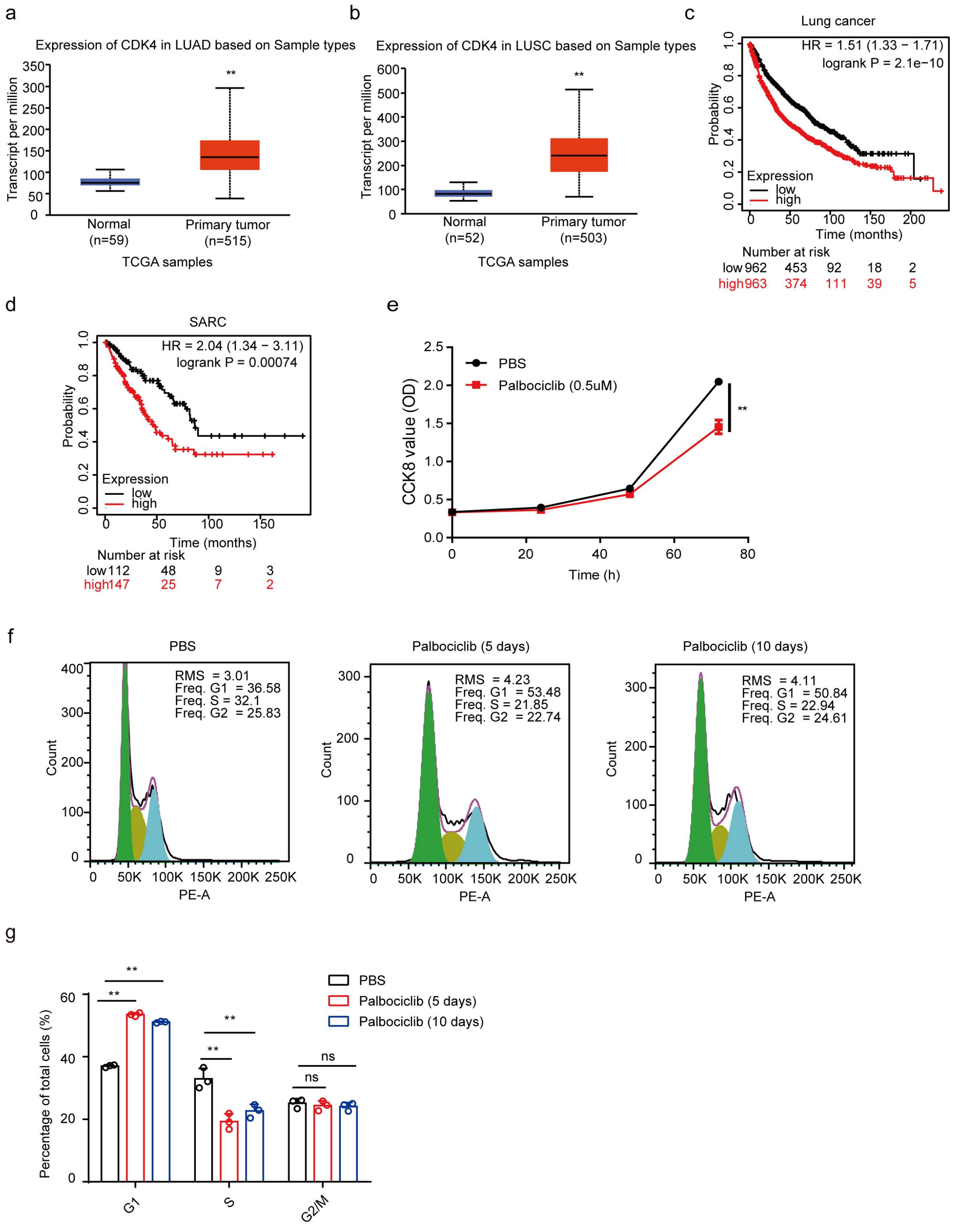

Supplementary Figure 1. High expression of CDK4 negatively correlates with patient survival rates and immune cell infiltration

a,b mRNA expression analysis of CDK4 between adjacent and cancerous tissue in LUAD patients (**a**) and LUSC patients (**b**) . $**p < 0.01$. **c, d** Graph generated from the Kaplan-Meier Plotter database showing the prognostic values of *Cdk4* in patients with lung cancer (**c**) and sarcoma (**d**). **e** Cell proliferation of MCA205 cells treated with PBS or palbociclib (0.5uM) for 10 days detected by CCK8 assay. **f, g** Cell cycle analysis of MCA205 cells treated with PBS, palbociclib (0.5uM) for 5 days and for 10 days detected with flow cytometry (**f**). Quantification of the

percentage of cells in different cell cycle phage (**g**). Data are representative of three independent experiments and presented as mean ± SD. Statistical significance was analyzed by unpaired Student's *t*-test. ns, no significant, $^{**}p < 0.01$.

Supplementary Figure 2

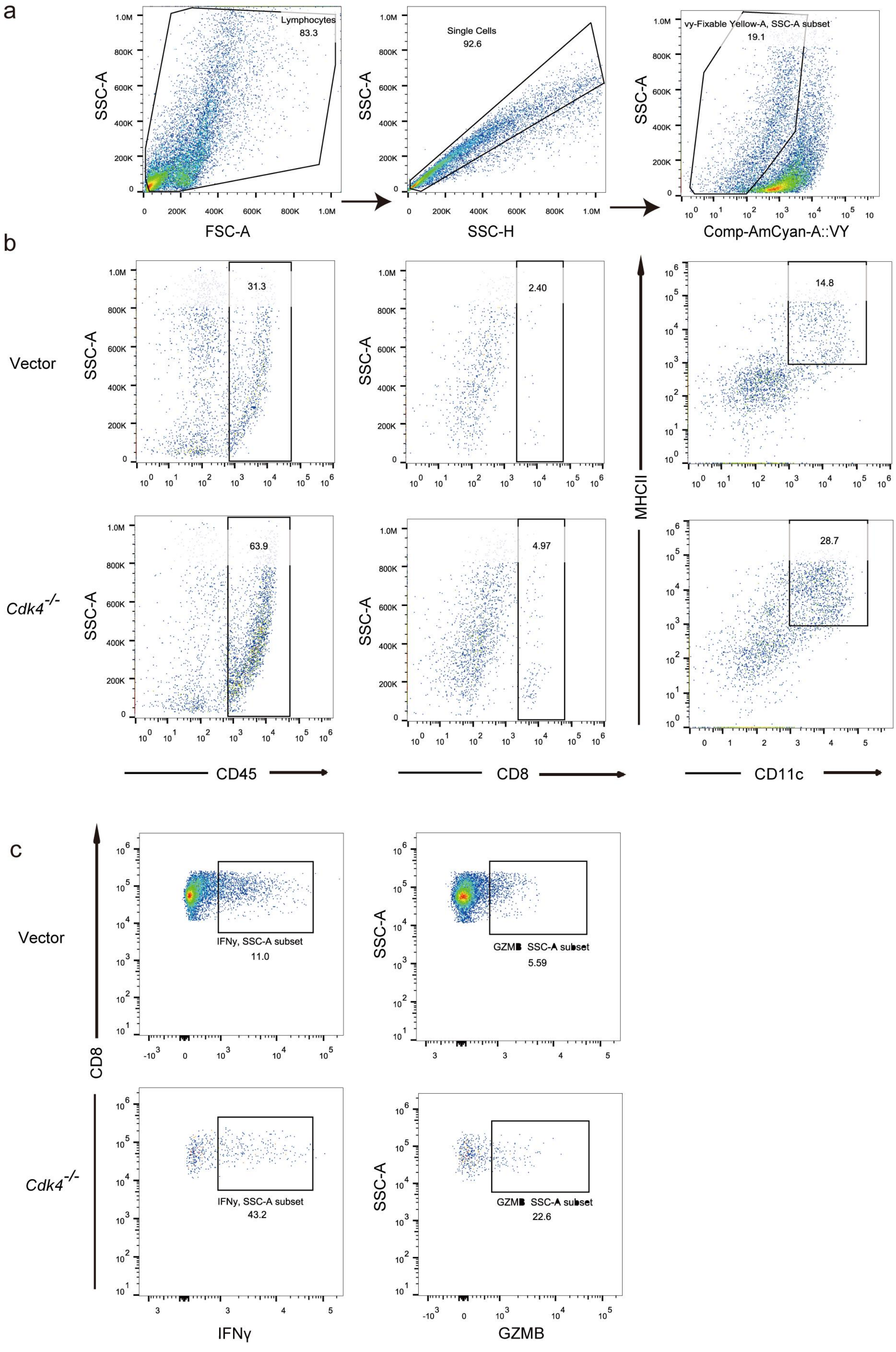

Supplementary Figure 2. ***Cdk4*-deficiency induced immune cell infiltration**

a-c Representative scatterplots of gated immune cells shown in Fig. 2**c**. Vector or *Cdk4*$^{-/-}$ MCA205 tumor cells (2 × 10^6/mouse) were transplanted subcutaneously on the right flank of C57BL/6N mice (n = 5). Tumors were harvested on day 10 after tumor transplantation and subjected to cytometric analysis. Gating process are shown in (**a**). Numbers indicate the percentage of $CD45^+$, $CD8^+$ and $CD11c^+$ cells population (**b**). IFN-γ and GZMB positive cells were gated from the CD8+ T cells population (**c**).

Supplementary Figure 3

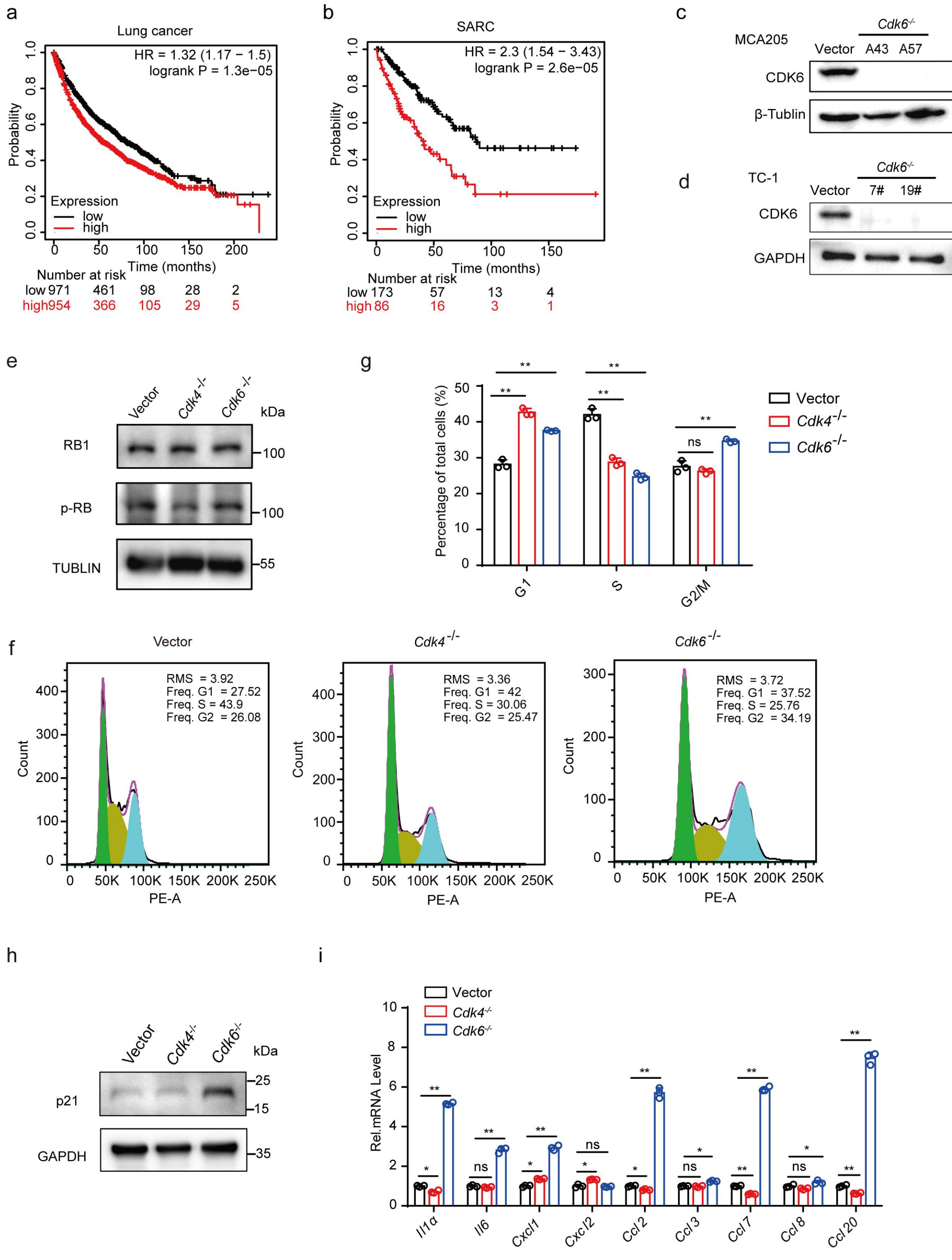

Supplementary Figure 3. CDK6 knockout induces cell cycle arrest and senescence

a, b Graph generated from the Kaplan-Meier Plotter database showing the prognostic values of *Cdk6* in patients with lung cancer (**a**) and sarcoma (**b**). **c** The success of Cdk6 knockout in MCA205 cell line was validated by Western Blot assay. **d** The success of Cdk6 knockout in TC1 cell line was validated by Western Blot assay. **e** Protein expression of RB1 and p-RB in vector, *Cdk4*$^{-/-}$ and *Cdk6*$^{-/-}$ MCA205 cells determined by Western blot assay. **f,g** Cell cycle analysis of vector, *Cdk4*$^{-/-}$ and *Cdk6*$^{-/-}$ MCA205 cells detected with flow cytometry (**f**). Quantification

of the percentage of cells in different cell cycle phage (**g**). **h** Protein expression of p21 in vector, *Cdk4*$^{-/-}$ and *Cdk6*$^{-/-}$ MCA205 cells determined by Western blot assay. i mRNA expression of *Il1α*, *Il6*, *Cxcl1*, *Cxcl2*, *Ccl2*, *Ccl3*, *Ccl7*, *Ccl8* and *Ccl20* in vector, *Cdk4*$^{-/-}$ and *Cdk6*$^{-/-}$ MCA205 cells determined by qPCR assay. Data are representative of three independent experiments and presented as mean ± SD. Statistical significance was analyzed by unpaired Student's *t*-test. ns, no significant, $*p < 0.05$, $**p < 0.01$.

Supplementary Figure 4

Figure 1c and d

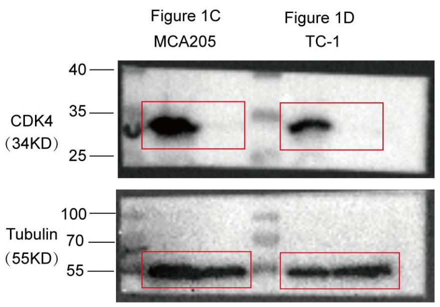

Figure 1e

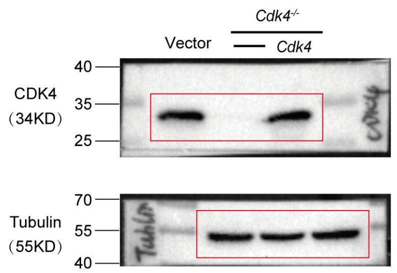

Figure 3f

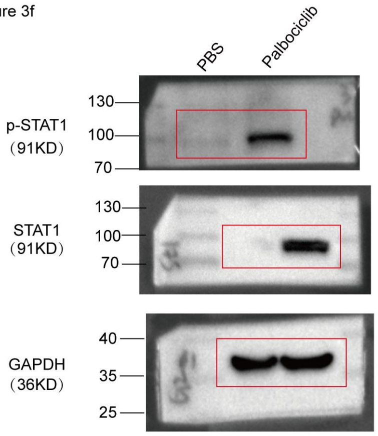

Figure 3g

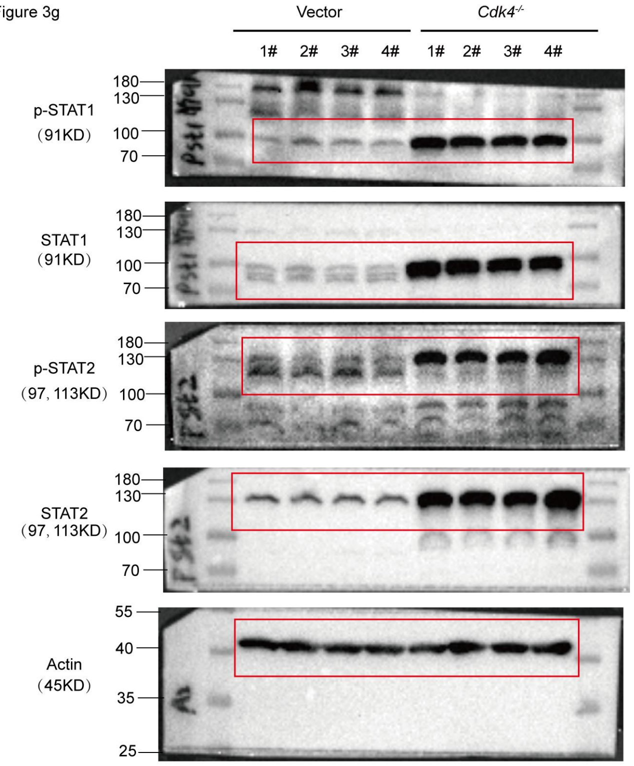

Figure 4a

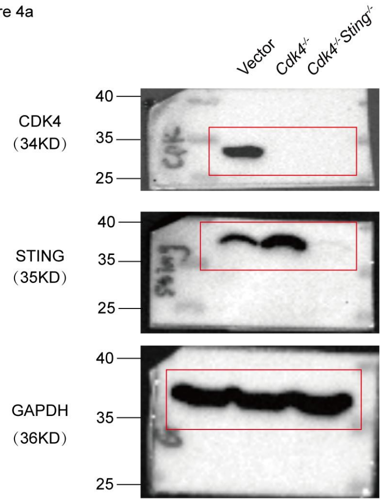

Figure 4b

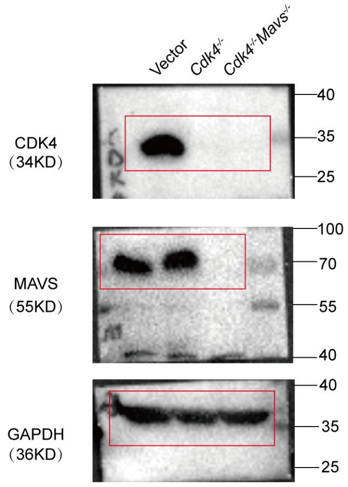

Supplementary Figure 4—Continued

Figure 4e

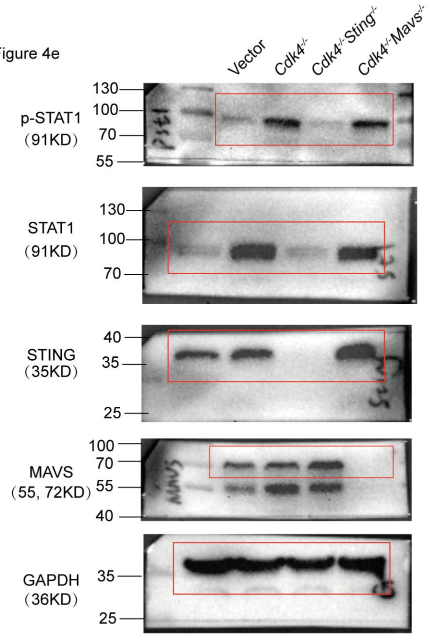

Figure 4i

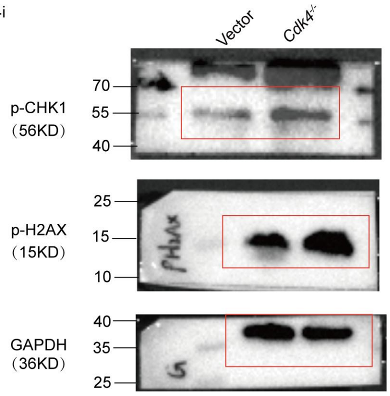

Figure 6a

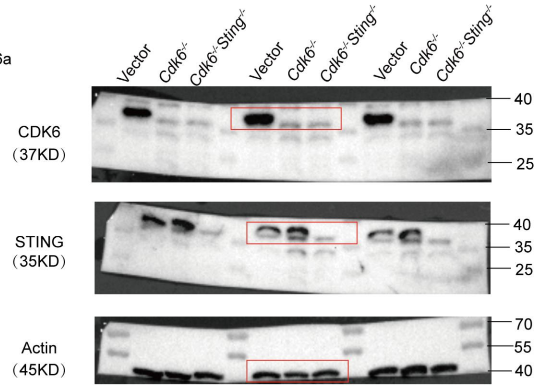

Figure 5l

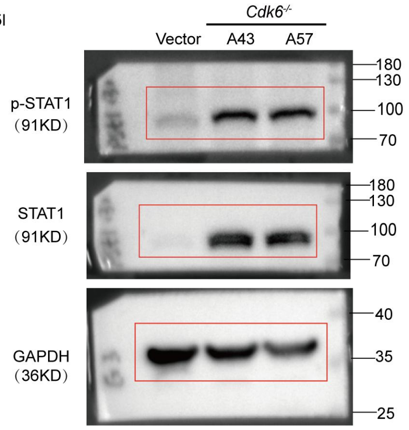

Figure 6b

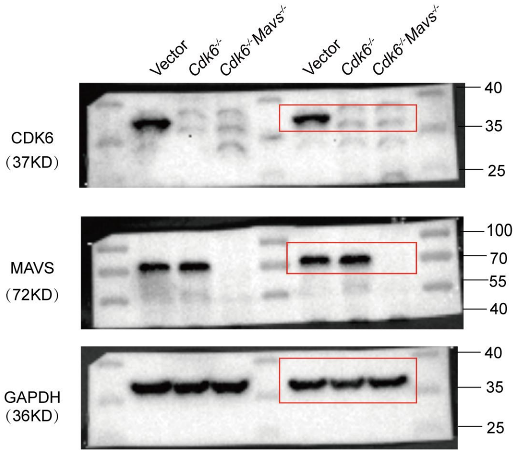

Figure 6c

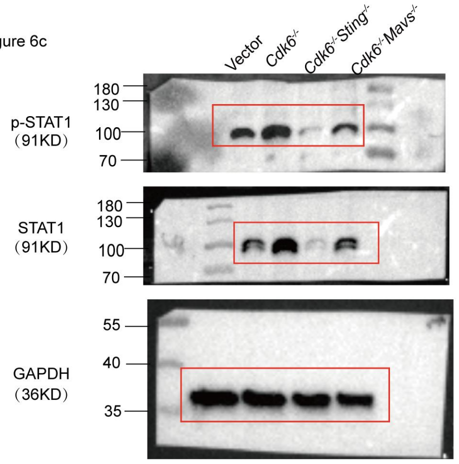

Supplementary Figure 4—Continued

Figure 6h

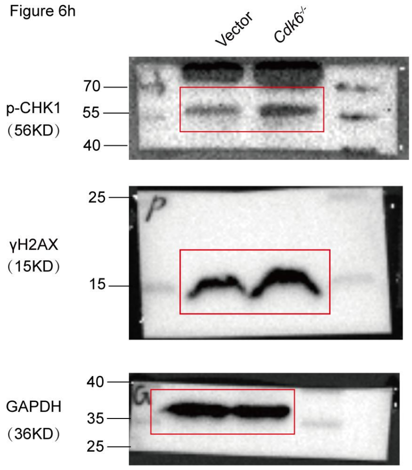

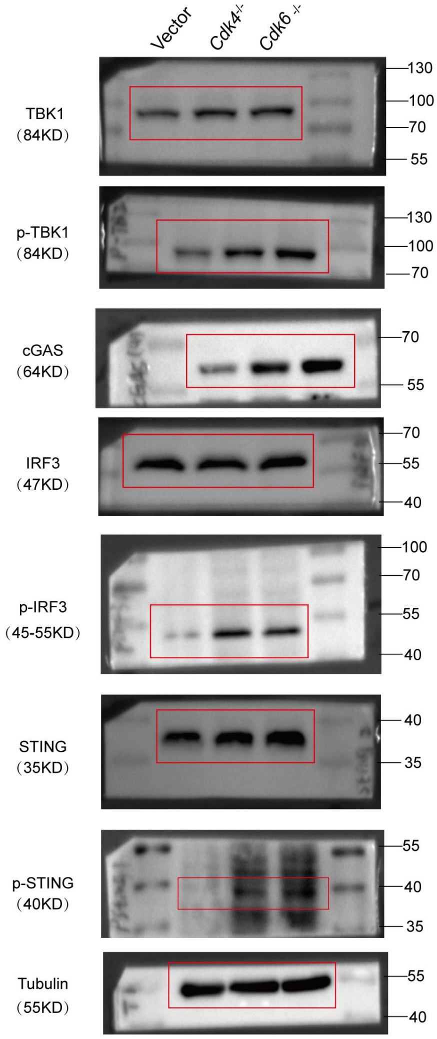

Figure 7b

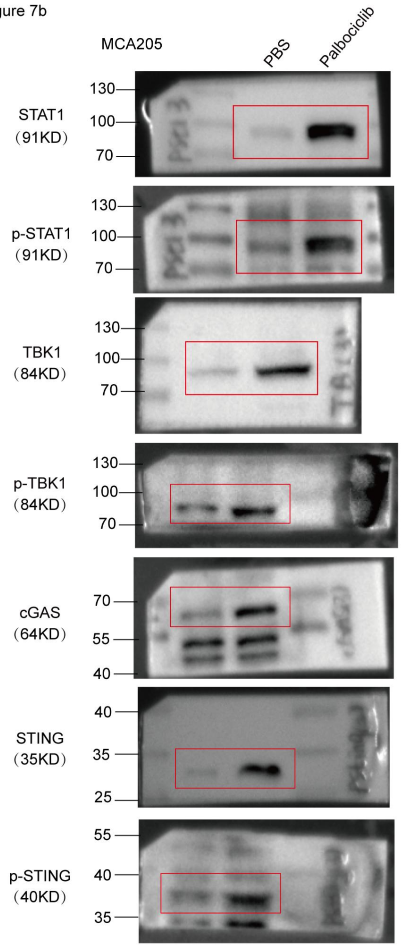

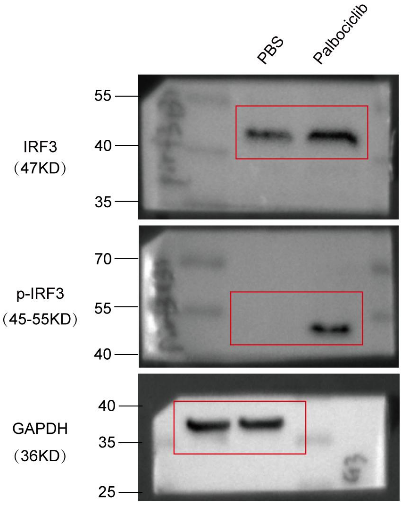

Supplementary Figure 4—Continued

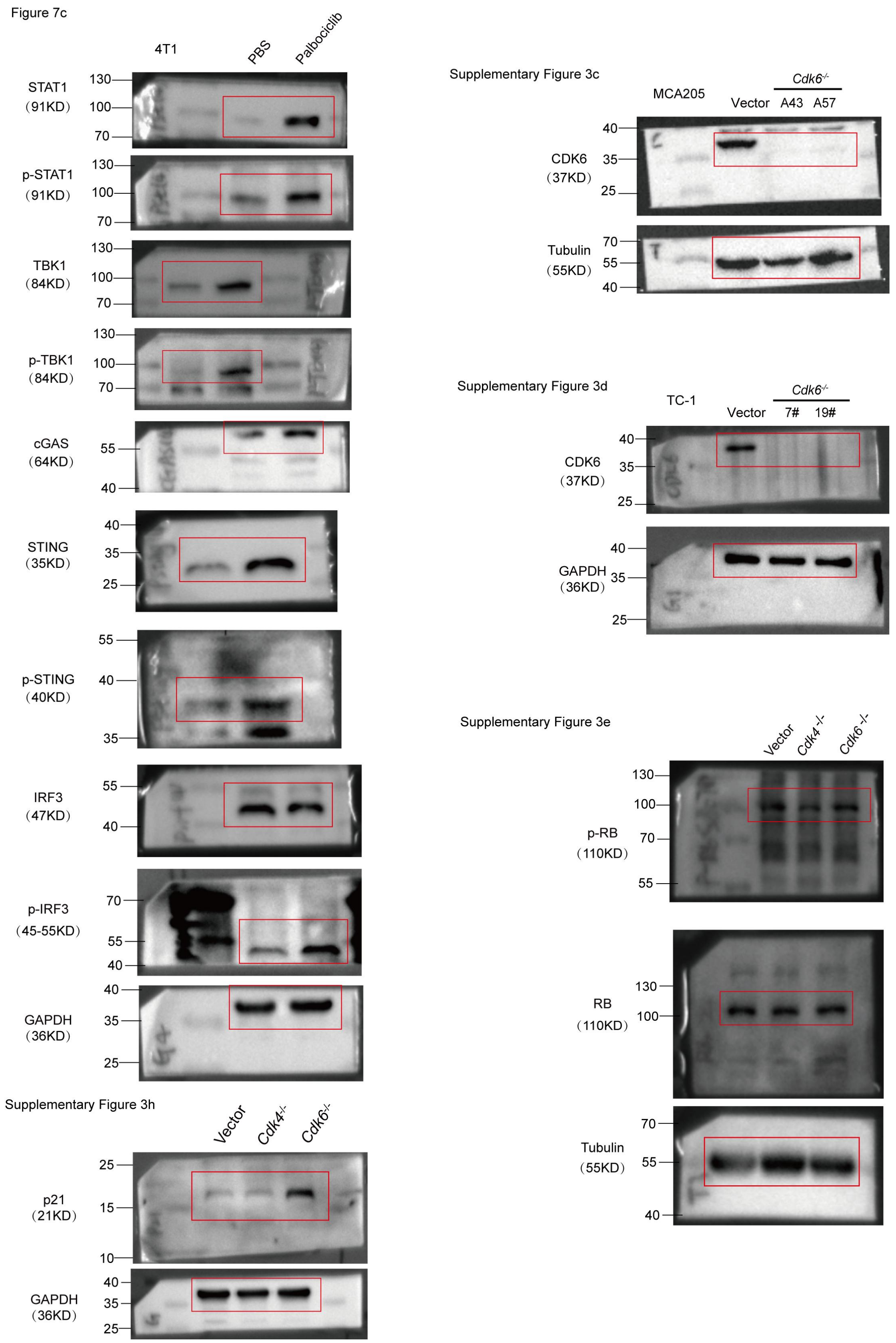

Supplementary Figure 4. Unedited and uncropped Western blots of all the indicated figures in main figures and supplementary figures.

Supplementary Table 1. Sequence of Guide RNA (sgRNA) oligonucleotides.

Gene	Forward	Reverse
Cdk4	CACCGCTCACCTTAATGGTCTCAAC	AAACGTTGAGACCATTAAGGTGAGC
Cdk6	CACCGAGTTCAGACGTGGATCAACT	AAACAGTTGATCCACGTCTGAACTC
Sting	CACCGCGGCAGTTATTTCGAGACTC	AAACGAGTCTCGAAATAACTGCCGC
Mavs	CACCGCCTACAGGCAGATCGGGAAC	AAACGTTCCCGATCTGCCTGTAGG C

Supplementary Table 2. Primers designed for real-time PCR.

Gene	Forward	Reverse
Gapdh	AGGTCGGTGTGAACGGATTTG	TGTAGACCATGTAGTTGAGGTCA
Stat1	TCACAGTGGTTCGAGCTTCAG	GCAAACGAGACATCATAGGCA
Stat2	TCCTGCCAATGGACGTTCG	GTCCCACTGGTTCAGTTGGT
Isg15	GGTGTCCGTGACTAACTCCAT	TGGAAAGGGTAAGACCGTCCT
Ifi204	AAAGGAGCCTGCTAAGGAAGA	CGTTCACATCAGAGACACAGGA
Ifit1	TGCTCCACTACTCCCCAACAT	GCTCCTACTGACCTCAGGTAAC
Il1α	CGAAGACTACAGTTCTGCCATT	GACGTTTCAGAGGTTCTCAGAG
Il6	TAGTCCTTCCTACCCCAATTTCC	TTGGTCCTTAGCCACTCCTTC
Cxcl1	CTGGGATTCACCTCAAGAACATC	CAGGGTCAAGGCAAGCCTC
Cxcl2	CCAACCACCAGGCTACAGG	GCGTCACACTCAAGCTCTG
Ccl2	TTAAAAACCTGGATCGGAACCAA	GCATTAGCTTCAGATTTACGGGT
Ccl3	TTCTCTGTACCATGACACTCTGC	CGTGGAATCTTCCGGCTGTAG
Ccl7	GCTGCTTTCAGCATCCAAGTG	CCAGGGACACCGACTACTG
Ccl8	TCTACGCAGTGCTTCTTTGCC	AAGGGGGATCTTCAGCTTTAGTA
Ccl20	GCCTCTCGTACATACAGACGC	CCAGTTCTGCTTTGGATCAGC
Mcm2	ATCCACCACCGCTTCAAGAAC	TACCACCAAACTCTCACGGTT
Mcm3	AGCGCAGAGAGACTACTTGGA	GCGGTTAGCCCTCTTTTCATTC
Mcm4	GAGGAAAGCAGGTCGTCACC	AGGGCTGGAAAACAAGGCATT
Mcm5	CAGAGGCGATTCAAGGAGTTC	CGATCCAGTATTCACCCAGGT
Pola2	TTTGTCCCGTCTCTGAGGGAT	CAGCTCGGAGAAGGTGAAAGG

Polg2	GAGGCGCTGGTAGACCTCT	GAGGCTAGGTTCTTTCGTAACTC
Poln	TATGTCTGCTATGCGTTCAGGT	TGAGTCTCCTCATGTTCAGCTA
