# Supplementary material for: DNA damage induced by CDK4 and CDK6 blockade triggers anti-tumor immune responses through cGAS-STING pathway
Source: Commun Biol. 2023 Oct 13;6:1041. doi: 10.1038/s42003-023-05412-x (PMC10575937; doi:10.1038/s42003-023-05412-x)
Supplement: Supplementary file 5 — Reporting Summary [file 42003_2023_5412_MOESM5_ESM.pdf]

nature portfolio

Corresponding author(s): *Double-anonymous peer review submissions: write DAPR and your manuscript number here instead of author names.*

Last updated by author(s): *YYYY-MM-DD*

Reporting Summary

Nature Portfolio wishes to improve the reproducibility of the work that we publish. This form provides structure for consistency and transparency in reporting. For further information on Nature Portfolio policies, see our Editorial Policies and the Editorial Policy Checklist.

Statistics

For all statistical analyses, confirm that the following items are present in the figure legend, table legend, main text, or Methods section.

n/a	Confirmed	
☐	☒	The exact sample size (*n*) for each experimental group/condition, given as a discrete number and unit of measurement
☐	☒	A statement on whether measurements were taken from distinct samples or whether the same sample was measured repeatedly
☐	☒	The statistical test(s) used AND whether they are one- or two-sided<br>*Only common tests should be described solely by name; describe more complex techniques in the Methods section.*
☒	☐	A description of all covariates tested
☒	☐	A description of any assumptions or corrections, such as tests of normality and adjustment for multiple comparisons
☐	☒	A full description of the statistical parameters including central tendency (e.g. means) or other basic estimates (e.g. regression coefficient) AND variation (e.g. standard deviation) or associated estimates of uncertainty (e.g. confidence intervals)
☐	☒	For null hypothesis testing, the test statistic (e.g. F, t, r) with confidence intervals, effect sizes, degrees of freedom and P value noted<br>*Give P values as exact values whenever suitable.*
☒	☐	For Bayesian analysis, information on the choice of priors and Markov chain Monte Carlo settings
☒	☐	For hierarchical and complex designs, identification of the appropriate level for tests and full reporting of outcomes
☒	☐	Estimates of effect sizes (e.g. Cohen's *d*, Pearson's *r*), indicating how they were calculated

Our web collection on statistics for biologists contains articles on many of the points above.

Software and code

Policy information about availability of computer code

Data collection	Clinical data were obtained from UALCAN database, Kaplan-Meier Plotter database and Timer 2.0 website in this study. Flow cytometry was conducted by Attune NxT Flow Cytometer (Thermo Fisher Scientific) and BD LSRFortessa. The western blot membranes were scanned with the ChemiDoc XRS+ system (Bio-Rad, USA). QPCR was detected using the LightCycler480II Real-Time PCR System (Roche). Image staining were captured with confocal microscope (Leica TCS SP8). Elispot plates were scanned using CTL ImmunoSpot S6 Analyzers (CTL and CTL Analyzers, LLC). ELISA data were obtained from SpectraMax device (Molecular Devices).
Data analysis	Gene Ontology analysis for RNA-seq was performed with David website (https://david.ncifcrf.gov/) and GSEA analysis was performed with GSEA 4.1.0 software. The western blot membranes were scanned with the ChemiDoc XRS+ system (Bio-Rad, USA) and analyzed with Image lab software. Flow cytometry was conducted by Attune NxT Flow Cytometer (Thermo Fisher Scientific) and analyzed with flowjo software. QPCR was detected using the LightCycler480II Real-Time PCR System (Roche) and analyzed with GraphPad Prism 7 software. Image staining was analyzed with image J software.

For manuscripts utilizing custom algorithms or software that are central to the research but not yet described in published literature, software must be made available to editors and reviewers. We strongly encourage code deposition in a community repository (e.g. GitHub). See the Nature Portfolio guidelines for submitting code & software for further information.

Data

Policy information about availability of data

All manuscripts must include a data availability statement. This statement should provide the following information, where applicable:

- Accession codes, unique identifiers, or web links for publicly available datasets
- A description of any restrictions on data availability
- For clinical datasets or third party data, please ensure that the statement adheres to our policy

Clinical datasets were from UALCAN database(https://ualcan.path.uab.edu/index.html), Kaplan-Meier Plotter database(https://kmplot.com/analysis/) and Timer 2.0 website(http://timer.cistrome.org/). Raw RNA-seq data are available in NCBI database(https://www.ncbi.nlm.nih.gov/) with the access number PRJNA893858 and in CNCB(https://www.cncb.ac.cn/) with the access number HRA003815.

Research involving human participants, their data, or biological material

Policy information about studies with human participants or human data. See also policy information about sex, gender (identity/presentation), and sexual orientation and race, ethnicity and racism.

Reporting on sex and gender	Only female were involved in this study because we collected human breast cancer patients' samples.
Reporting on race, ethnicity, or other socially relevant groupings	There are no race, ethnicity or other socially relevant groupings.
Population characteristics	This study recruited 125 breast cancer patients for immunohistochemical (IHC) analysis and 10 breast cancer patients (5 with CDK4/6 inhibitors treatment and 5 without) for RNA-seq analysis from the Affiliated Tumor Hospital of Nantong University. The patients information could be seen in Table 1 and Table 2.
Recruitment	Participants were recruited by physician. All participants were after mastectomy.
Ethics oversight	The study was conducted according to the principles of the Declaration of Helsinki and approved by the Human Research Ethics Committee of the Affiliated Tumor Hospital of Nantong University with the ethic number of 2022-039.

Note that full information on the approval of the study protocol must also be provided in the manuscript.

Field-specific reporting

Please select the one below that is the best fit for your research. If you are not sure, read the appropriate sections before making your selection.

☒ Life sciences ☐ Behavioural & social sciences ☐ Ecological, evolutionary & environmental sciences

For a reference copy of the document with all sections, see nature.com/documents/nr-reporting-summary-flat.pdf

Life sciences study design

All studies must disclose on these points even when the disclosure is negative.

Sample size	For mouse model and in vivo study, we have at least three mice in one group, normally five mice in a group. In vitro study, we have repeated each experiment for at least three times. Human samples were collected as the largest amount we can get from the Affiliated Tumor Hospital of Nantong University.
Data exclusions	No data were excluded from the analyses.
Replication	All experiments were conducted for 2-3 times and get similar results.
Randomization	All samples and mice were randomly divided into experimental groups.
Blinding	Investigators were not blinded to group allocation during data collection and analysis because we value tumor size and gave different treatments to different groups.

Reporting for specific materials, systems and methods

We require information from authors about some types of materials, experimental systems and methods used in many studies. Here, indicate whether each material, system or method listed is relevant to your study. If you are not sure if a list item applies to your research, read the appropriate section before selecting a response.

Materials & experimental systems

n/a	Involved in the study
☐	☒ Antibodies
☐	☒ Eukaryotic cell lines
☒	☐ Palaeontology and archaeology
☐	☒ Animals and other organisms
☒	☐ Clinical data
☒	☐ Dual use research of concern
☒	☐ Plants

Methods

n/a	Involved in the study
☒	☐ ChIP-seq
☐	☒ Flow cytometry
☒	☐ MRI-based neuroimaging

Antibodies

Antibodies used

Anti-CDK4 antibody(abcam, cat:ab199728, clone name:EPR17525, lot:GR207212-7)
Anti-CDK6 antibody(abcam, cat:ab241554, clone name:98D, lot:GR3321537-4)
anti-STING antibody(CST, cat:13647S, clone name: D2P2F, lot:5)
Anti-MAVS antibody(CST, cat:4983S, lot:3)
Anti-Phospho-STAT1 antibody(CST, cat:9167, clone name:58D6, lot:5)
Anti-STAT1 antibody(CST, cat:14994, clone name:D1K9Y, lot:26)
Anti-Phospho-STAT2 (CST, cat:a88410S, clone name:D3P2P, lot:4)
Anti-STAT2 antibody(CST, cat:72604S, clone name: D9J7L, lot:4)
Anti-β-Actin antibody(CST, cat:8457S, clone name: D6A8, lot:8)
Anti-vH2AX antibody(CST, cat:9718, clone name:20E3, lot:17)
Anti-Phospho-CHK1 antibody(CST, cat:12302, clone name:D12H3, lot:18)
Anti-α/β-Tublin antibody(CST, cat:2148S, lot:8).
For flow cytometry , we used:
CD45.2 (BioLegend, 109828, clone 104)
CD8 (BioLegend, 100723, clone: 53-6.7)
CD11b (BioLegend, 101228, clone: M1/70)
CD11c (BioLegend, 117318, clone: N418)
IA/IE (BioLegend, 107616, clone: M5/114.15.2)
anti-granzyme B (BioLegend, 515406, clone: GB11)
anti-IFN-γ(BioLegend, 505813, clone: XMG1.2).

Validation

The validation statements are available in the manufactures' websites. And their specificity was further verified by the size and location of the bands in western blot membranes.

Eukaryotic cell lines

Policy information about cell lines and Sex and Gender in Research

Cell line source(s)

TC1 and HEK293T cell lines were obtained from Cell Resource Center, Institute of Basic Medical Sciences, Chinese Academy of Medical Sciences (Beijing, China). MCA205 was obtained from Dr. S. A. Rosenberg (NCI, Bethesda, MD).

Authentication

Authentication of cell lines with short tandem repeat DNA profiles was performed every year with Procell Life Science & Technology Co.

Mycoplasma contamination

All cell lines were tested negatively for mycoplasma contamination.

Commonly misidentified lines
(See ICLAC register)

There are no misidentified cell lines in this study.

Animals and other research organisms

Policy information about studies involving animals; ARRIVE guidelines recommended for reporting animal research, and Sex and Gender in Research

Laboratory animals

6-8-week female mice were used for all animal experiments. C57BL/6 and athymic nude BALB/c mice (nu/nu) were purchased from Beijing Vital River Company. Ifnar1-/- C57BL/6 mice were purchased from Model Animal Research Center of Nanjing University. NOD.Cg-Prkdcscid IL2rgtm1Wjl/SzJ mice (NSG) were purchased from Beijing Biocytogen Company. Animal experimental protocols were approved by the Institutional Animal Care and Use Committee (IACUC) of Suzhou Institute of System Medicine.

Wild animals

The study did not involve wild animals.

Reporting on sex

In immunological experiments, female mice are usually selected because androgen level of male mice can affect experimental results.

Field-collected samples

The study did not involve samples collected from the field.

Ethics oversight	Animal experimental protocols were approved by the Institutional Animal Care and Use Committee (IACUC) of Suzhou Institute of System Medicine.

Note that full information on the approval of the study protocol must also be provided in the manuscript.

Flow Cytometry

Plots

Confirm that:

☒ The axis labels state the marker and fluorochrome used (e.g. CD4-FITC).

☒ The axis scales are clearly visible. Include numbers along axes only for bottom left plot of group (a 'group' is an analysis of identical markers).

☒ All plots are contour plots with outliers or pseudocolor plots.

☒ A numerical value for number of cells or percentage (with statistics) is provided.

Methodology

Sample preparation	Mice were euthanized by CO2 inhalation and MCA205 tumor tissues were took out and cut into small pieces with surgical scissors in serum-free RPMI 1640 media. Then tumor tissues were digested with DNase I (Sigma, 260913-10 MU) and Liberase TL (2 μg/mL, Roche, 05401020001) at 37 ℃ incubator for at least 1h. The cell suspension went through a 70 μm filter (ThermoFisher Scientific) and pelleted by centrifuge at 1500 rpm for 5 min. Then pelleted cells were washed and resuspend in PBS containing dye for flow cytometric analysis. Live cells were strained by fluorescence-labeled antibodies against vivid yellow (Invitrogen, #L34959). Cell surface markers were stained by mouse specific antibodies CD45.2 (BioLegend, 109828), CD8 (BioLegend, 100723), CD11b (BioLegend, 101228), CD11c (BioLegend, 117318), IA/IE (BioLegend, 107616) at 4 ℃ for 30 min. For GZMB and IFN-γ staining, cells were fixed with infixation/permeabilization kit (BD Bioscience, 554714) at 4 ℃ for 30 min, then stained with anti-granzyme B (BioLegend, 515406) and anti-IFN-γ(BioLegend, 505813). All these antibodies were diluted by 1:100 using PBS. Stained cells were washed by PBS once and then conducted using BD LSRFortessa and analyzed with flowjo software (Tree Star, Inc., Ashland, OR, USA).
Instrument	BD LSRFortessa and Attune NxT Flow Cytometer (Thermo Fisher Scientific)
Software	FACS data were collected using BD LSRFortessa a,Attune NxT Flow Cytometer (Thermo Fisher Scientific), and Flowjo software(Tree Star) was used to analysis the data.
Cell population abundance	We removed red cells with red cell lysis buffer to ensure that cells for flow cytometry were tumor cells and ilymphocytes . And we gate lymphocytes according to the FSC and SSC value.
Gating strategy	Gate cells exclude dead cells and tumor cells based on cell size, then gate vivid yellow negative cells as live cells. Then gate CD45.2+ cells and gate CD45.2+CD3+ cells, then gate CD45.2+CD3+CD8+ as CD8+ T cells and CD45+IA/IE+CD11c+ cells as DCs. For effector T cell gating, we gate CD45+CD3+CD8+IFNγ+ and CD45+CD3+CD8+GZMB+ cells.

☒ Tick this box to confirm that a figure exemplifying the gating strategy is provided in the Supplementary Information.
